# Molecular Analysis of Fluoroquinolone-resistant *Salmonella* Paratyphi A Isolate, India

**DOI:** 10.3201/eid1203.050560

**Published:** 2006-03

**Authors:** Satheesh Nair, Madhulika Unnikrishnan, Keith Turner, Subash Chandra Parija, Carol Churcher, John Wain, Belgode Narasimha Harish

**Affiliations:** * The Wellcome Trust Sanger Institute, Cambridgeshire, United Kingdom;; † Jawaharlal Institute of Postgraduate Medical Education and Research, Pondicherry, India

**Keywords:** Fluoroquinolones, Salmonella enterica serovar Paratyphi A, DNA gyrase, Topoisomerase IV, antibiotic resistance

## Abstract

*Salmonella enterica* serovar Paratyphi A is increasingly a cause of enteric fever. Sequence analysis of an Indian isolate showed a unique strain with high-level resistance to ciprofloxacin associated with double mutations in the DNA gyrase subunit *gyr*A (Ser83→Phe and Asp87→Gly) and a mutation in topoisomerase IV subunit *par*C (Ser80→Arg).

*Salmonella enterica* serovar Paratyphi A is the second most common cause of enteric fever after *S*. Typhi. Approximately 0.25 *S*. Paratyphi A infections (paratyphoid fever) occur for each *S*. Typhi infection (typhoid fever) (*1*). Given global estimates of >21 million cases of typhoid fever in the year 2000, >5 million cases per year of *S*. Paratyphi A probably occur. Paratyphoid fever is a major clinical problem in India, but large outbreaks were not reported until 1996 (*2*). Elsewhere, in southern China for example, extensive outbreaks are also occurring (*3*).

Since 1998, plasmid-mediated multidrug resistance in *S*. Paratyphi A, associated with chromosomally mediated reduced susceptibility to ciprofloxacin, has caused concern (*4*). Reduced susceptibility to fluoroquinolones results in a poor response of salmonellosis patients to treatment and may allow prolonged bacterial shedding (*5*). Rising resistance to fluoroquinolones is likely to be driving an increase in cases of paratyphoid fever in regions where fluoroquinolones are used empirically to treat enteric fever. We must monitor the emergence of resistance in this enteric pathogen to differentiate between the acquisition of resistance during treatment (mutations occurring in different bacterial strains) or clonal expansion of a successful strain by person-to-person spread (identical mutations associated with a single strain). To do so, we need to describe the molecular basis of resistance and the genotype of the resistant strains. High-level ciprofloxacin-resistant *S*. Paratyphi A (MIC 8 μg/mL) is present in India (*6*) and Japan (MIC >128 μg/mL) (*7*). However, because S. Typhi, the major cause of enteric fever in India, has not yet developed high-level resistance to fluoroquinolones, enteric fevers are often treated empirically with fluoroquinolones. If this trend continues, fluoroquinolone-resistant strains of *S*. Paratyphi A are almost certain to become a major cause of enteric fever in many areas.

We analyzed, by DNA sequencing, the DNA gyrase and topoisomerase IV genes of the first reported highly fluoroquinolone-resistant *S*. Paratyphi A isolate (*6*). We looked at the full coding sequence, including the quinolone resistance–determining region (QRDR), of both subunits of DNA gyrase and topoisomerase IV for mutations associated with resistance to fluoroquinolones. We also used multilocus sequence typing (MLST) to confirm the identity of the isolate.

## The Study

The strain described here (Pond1) was first isolated in Pondicherry, India, in November 2002 from the blood of a 23-year-old man admitted with fever and with no history of having received antimicrobial chemotherapy (*6*). The isolate was resistant to ciprofloxacin and nalidixic acid, and the MIC of ciprofloxacin was 8 mg/L. It was sensitive to all other antimicrobial drugs tested by disk diffusion: ampicillin, chloramphenicol, cotrimoxazole, gentamicin, and ceftriaxone. Repeat testing showed that zones of inhibition indicating susceptibility were seen around both ofloxacin (17 mm) and ciprofloxacin (21 mm) 5-μg disks; however, in light of the ciprofloxacin MIC and resistance to nalidixic acid, Pond1 was considered resistant to fluoroquinolones. No similar isolates have been seen in this area since the initial report, although the total number of paratyphoid fever cases has increased.

Polymerase chain reaction (PCR) amplification (Table 1) and direct DNA sequencing of both strands of the full length of gyrase (*gyrA* and *gyrB*) and topoisomerase IV (*parC* and *parE*) subunit genes was performed with an ABI Prism dye terminator cycle sequencing kit (Perkin Elmer, Foster City, CA, USA) on an ABI 3730 automated sequencer. Results showed 3 mutations: 2 in *gyrA* and 1 in *parC*. A comparison of these mutations with those previously described in fluoroquinolone-resistant *S*. Paratyphi A is shown in Table 2. A rise of fluoroquinolone resistance over time is apparent, and although the point mutations do not fully explain the MIC data, we noted general associations: a single mutation in *gyrA* is always associated with resistance to nalidixic acid and reduced susceptibility to ciprofloxacin and ofloxacin, and a double mutation in *gyrA* is always accompanied by mutations in *parC* and is associated with high-level (>4 μg/mL) resistance. Although this sample is limited, heterogeneity in mutations found at all sites suggests that this finding is not the result of a single successful strain's sequentially acquiring mutations but rather that resistance is arising in several different strains.

**Table 1:** Primer sequences for amplification of topoisomerases

Primer	Sequence 5´–3´	Amplicon	Gene (coding sequence length)
gyrA7 (F)	5´ GGGTCGACTGATTATGGTTTATGCCTCC 3´	3199	2637
gyrA25 (R)	5´ GAGACTTTCAGCGTAGTTCG 3´		
gyrB1 (F)	5´ TTGCCTCTGAACTTGACGATGC 3´	2762	2415
gyrB9 (R)	5´ GAAGTCGCTGACCTGCTCAC 3´		
parC3 (F)	5´ CGATTTTCCGGTCTTCTTCCAG 3´	2616	2259
parC10 (R)	5´ GCAATGCACGAATAAACAACGG 3´		
parE3 (F)	5´ CCTGATCTGGCTACTGCAACAG 3´	2183	1893
parE8 (R)	5´ ATGCGCAAGTGTCGCCATCAG 3´		

**Table 2:** Presence of *qnr* and mutations in DNA gyrase and topoisomerase IV genes of *Salmonella enterica* serovar Paratyphi A strains associated with decreased susceptibility or resistance to ciprofloxacin*

Country	Year	MIC (μg/mL)					
		Cp	Nal	*qnr*	*gyrA*	*parC*	Reference
India	1999	0.38	>256	ND	Ser83→Phe	ND	(*8*)
Bangladesh	1999	0.5	>256	ND	Asp87→Gly	ND	(*8*)
India	1999	0.5	>256	ND	Ser83→Phe	ND	(*8*)
Hong Kong	2000	0.5	>256	ND	Ser83→Tyr	NM	(*9*)
Japan	2002	>128	>256	ND	Ser83→Phe, Asp87→Asn	Glu84→Lys	(*7*)
India	2002	8	>256	NP	Ser83→Phe, Asp87→Gly	Ser80→Arg†	This study

*No mutations were detected in *gyrB* and *parE*. Cp, ciprofloxacin; Nal, nalidixic acid; ND, not determined; NP, not present; NM, no mutation within the quinolone resistance–determining region. †European Molecular Biology Laboratory accession no. AM050347.

## Conclusions

The QRDR within topoisomerases contains hotspots for mutations around the active site, which are associated with raised MIC values for fluoroquinolones (*10*). For *gyrA* from nalidixic acid–resistant *Salmonella* isolates, 2 mutations are most frequently observed in clinical isolates: Ser83→Phe and Ser83→Tyr (*8**,**9**,**11*). The association with resistance of mutations seen in *parC*, however, is less clear (*12*). Mutations in *parC* of gram-negative bacteria are usually within the QRDR at amino acids 80 and 84 (Ser 80→Ile, Glu 84→Gly, Lys). The first reported mutation was Thr57→Ser, and other mutations have been described: Asp 79→Ala, Gly 78→Asp (*9*). Each mutation, in *gyrA* or *parC*, can give rise to different MICs in different isolates, which means that other factors must also influence the resistance phenotype in *S*. Paratyphi A. The most likely cause is changes in expression levels of proteins involved with permeability barriers and efflux pumps. These changes could be the result of either point mutations in transcription promoters and regulators or downstream effects of mutations in topoisomerases. Known mechanisms of fluoroquinolone resistance that we were able to screen for include transmissible plasmidborne resistance and efflux pumps. The *qnr*-containing plasmid was not detected with PCR using primers 5´ GGG TAT GGA TAT TAT TGA TAA 3´ and 5´ CTA ATC CGG CAG CAC TAT TA 3´ (*13*) in Pond1, and sensitivity testing gave the same zone size around both tetracycline and chloramphenicol discs when compared with a nalidixic acid–susceptible isolate. This finding, combined with the ciprofloxacin MIC of 8 μg/mL, argues against the presence of multiple antimicrobial drug resistance efflux pumps. Thus the high-level resistance seen in Pond1 appears to be associated directly with 3 point mutations in the topoisomerase genes.

To confirm the identity of the isolate as *S*. Paratyphi A, we used MLST. The primer sequences and MLST data are available from the MLST database at the Max-Planck-Institut für Infektionsbiologie (http://web.mpiib-berlin.mpg.de/mlst/). Six of 7 sequenced loci matched exactly the previously described sequences, and 1 was a unique allele. This finding means that the isolate described here is within the clonal MLST group described as *S*. Paratyphi A but is a recognizable variant. This finding supports previous typing data that show very little variation (*14*); typing *S*. Paratyphi A is problematic because genomic restriction analyses (pulsed-field gel electrophoresis) of isolates from an outbreak are not always identical, and susceptible and resistant strains cannot be differentiated. For molecular epidemiologic studies to be carried out, several methods need to be used (*15*). A broader study of single base-pair differences between strains of *S*. Paratyphi A could provide a usable typing scheme.

Resistance in *S*. Paratyphi A populations must be monitored because the acquisition of resistance to fluoroquinolones, coupled with the reduction in *S*. Typhi by the use of typhoid-specific vaccination, may cause *S*. Paratyphi A to become the main cause of enteric fever. Disc susceptibility testing does not always detect resistance, and screening with nalidixic acid and MIC testing remains the method of choice. The isolate described here, Pond1, contains a unique combination of mutations that provides a way to track the spread of this strain of *S*. Paratyphi A.
